# The Real-World Experience of the Biosimilar (Grastofil^®^) to the Reference Biologic (Neupogen^®^) in Breast Cancer and Lymphoma: A Canadian Single-Centre Retrospective Study

**DOI:** 10.3390/curroncol29030115

**Published:** 2022-02-23

**Authors:** Gina Wong, Katie Wang, Mark Pasetka, Liying Zhang, Julia Lou, Habeeb Majeed, Jerome Flores, Emily Lam, Carlo DeAngelis

**Affiliations:** 1 Odette Cancer Centre, Sunnybrook Health Sciences Centre, University of Toronto, Toronto, ON M4N 3M5, Canada; wang.katie@hotmail.com (K.W.); mark.pasetka@sunnybrook.ca (M.P.); habeeb.majeed@sunnybrook.ca (H.M.); es3lam@uwaterloo.ca (E.L.); carlo.deangelis@sunnybrook.ca (C.D.); 2 Macrostat Inc., Toronto, ON L4B 4P4, Canada; zhangliege@gmail.com (L.Z.); louj14@mcmaster.ca (J.L.); 3 Department of Pharmacy, University Health Network, University of Toronto, Toronto, ON M5G 2C4, Canada; jerome.flores@mail.utoronto.ca

**Keywords:** biosimilar, breast cancer, lymphoma, primary prophylaxis, febrile neutropenia, retrospective study, Neupogen^®^ and Grastofil^®^

## Abstract

Febrile neutropenia (FN) is a common side effect of cytotoxic chemotherapy that may result in poor treatment outcomes. The short acting granulocyte colony stimulating factors (G-CSF) act to stimulate granulocytes to increase production of white blood cells. The filgrastim biosimilar is useful, as it may provide a cheaper and equally effective treatment to FN. This study explored the usage of the filgrastim biosimilar (Grastofil^®^) and the reference biologic (Neupogen^®^) in breast cancer and lymphoma patients. A retrospective chart review of patients receiving Grastofil^®^ from January 2017 to June 2019 or Neupogen^®^ for primary prophylaxis of FN from January 2013 to December 2017 was conducted. The endpoints included the incidence of FN and the occurrence of dose reduction (DR) and dose delay (DD). One hundred and fifty-three Grastofil^®^ patients were matched to 153 Neupogen^®^ patients. This cohort was further split into breast cancer (*n* = 275) and non-Hodgkin’s lymphoma (*n* = 31) cohorts. After adjusting for chemotherapy cycles, the biosimilar filgrastim was non-inferior to the reference biologic based on FN incidence in addition to related outcomes including DR and DD.

## 1. Introduction

Cancer patients undergoing treatment with myelosuppressive chemotherapy may be at risk of experiencing febrile neutropenia (FN) and its associated complications, which increase the risk of infections and negatively impact their chemotherapy treatment and overall quality of life [1]. FN is defined as an absolute neutrophil count (ANC) <1000/mm^3^ with a single temperature of greater than 38.3 °C (101 °F) or a sustained temperature of ≥38 °C (100.4 °F) for more than one hour (Common Terminology Criteria for Adverse Events, CTCAE) [2,3]. FN not only decreases a patient’s quality of life, but it is also associated with dose delays (DD) and dose reductions (DR) [4,5,6]. Any delay or reduction in chemotherapy treatment could lead to decreased treatment efficacy [6,7]. Furthermore, FN could lead to hospitalization and may negatively impact the health care system through increasing health care spending [8,9,10]. International guidelines recommend prophylactic use of G-CSF based on FN risk-determined by numerous patient- and disease-related factors [3,11,12]. Therefore, the use of granulocyte-colony stimulating factors (G-CSFs) for the primary prophylaxis of FN is an important supportive care that can aid in optimizing treatment delivery by reducing the severity and duration of neutropenia [5,13,14].

Filgrastim is a short-acting G-CSF that is administered daily throughout the duration of a patient’s chemotherapy treatment [3]. In a retrospective study by Weycker et al. based on 5477 patients, fewer days of filgrastim prophylaxis was associated with a higher risk of chemotherapy-induced neutropenia complications (CINC) (odds ratio (OR) = 2.35 for 1–3 days and OR = 1.93 for 4–6 days vs. 7+ days of filgrastim prophylaxis) [1].

G-CSFs are highly beneficial in reducing morbidity and mortality associated with chemotherapy-induced neutropenia, but like many drug products, G-CSFs are associated with health care expenditures [15]. The recent adoption and introduction of biosimilars offer lower-cost versions of previously approved reference biologics. Biosimilars undergo rigorous testing to be deemed bioequivalent, or molecularly similar in pharmacokinetics and pharmacodynamics, to the reference biologic [16,17]. The intent of biosimilars is to decrease the costs associated with high-priced biologics and their implementation, which can increase patient access to treatment and directly reduce health care costs [18,19]. Their implementation in several European countries has conferred significant cost savings [20]. Furthermore, the use of biosimilars can increase patient access to treatment in addition to directly reducing health costs. Currently, the implementation of various biosimilars has proved to reduce costs, whilst maintaining a similar safety and efficacy [19]. Numerous clinical trials and real-world data have compared the clinical outcomes of G-CSF biosimilars with their respective reference biologics for FN prophylaxis, which reported comparable effectiveness and safety [21].

Grastofil^®^ (Apobiologix, Toronto, ON, Canada) is a biosimilar filgrastim product to the reference biologic drug Neupogen^®^ (Amgen, Mississauga, ON, Canada) and was recommended by the Canadian Drug Expert Committee (CDEC), a pan-Canadian advisory body to Canadian Agency for Drugs and Technologies in Health (CADTH), and subsequently approved and listed by Health Canada to be indicated in a similar manner to Neupogen^®^. In order to gain this approval, the filgrastim biosimilar underwent rigorous testing and was proven to have similar pharmacokinetics, pharmacodynamics, and molecular integrity as the filgrastim biologic. Effective 22 December 2016, the Ontario Drug Benefit (ODB) program began funding Grastofil^®^ for the prevention and treatment of neutropenia associated with chemotherapy for eligible patients in Ontario, Canada [22]. Given the potential of providing significant cost savings compared with the cost of treatment with Neupogen^®^, beginning January 2017 at our centre, Grastofil^®^ replaced Neupogen^®^ for FN prophylaxis in new cancer patients starting myelosuppressive chemotherapy.

Despite the clinical data presented by CADTH supporting the use of Grastofil^®^, clinician and patient perspectives are still variable [23,24,25,26]. Therefore, the primary aim of this study was to assess the non-inferiority of the biosimilar Grastofil^®^ to the reference biologic Neupogen^®^ in terms of comparative safety and efficacy in real-world use as primary prophylaxis of FN in breast cancer and lymphoma patients.

## 2. Materials and Methods

### 2.1. Definition of Study Endpoints

The primary objective of this study was to assess the non-inferiority of the biosimilar Grastofil^®^ to its reference biologic Neupogen^®^. The primary endpoint was the incidence of FN events during chemotherapy treatment, which was comparatively assessed as the risk difference between the Grastofil^®^ and Neupogen^®^ cohorts. The secondary objective was to assess the clinical comparability of these drug products in terms of safety and efficacy. Secondary endpoints included FN event throughout the entire course of chemotherapy, length of hospitalization, and chemotherapy DR and DD incidence.

We defined the incidences of FN, DD, and DR as they are in clinical practice at our centre. Any reported event of FN during treatment and up to four weeks post-treatment was included in our analysis. DD was defined as a delay in treatment of 6 or more days, and DR was defined as any reduction in dosage in one or more chemotherapy agents in the regimen compared with baseline dosage.

### 2.2. Eligibility Criteria

All non-metastatic breast cancer and non-Hodgkin’s lymphoma patients who received Grastofil^®^ from January 2017 to June 2019 or Neupogen^®^ from January 2013 to December 2017 were screened. Only patients who were chemo-naïve, starting adjuvant/neoadjuvant or curative chemotherapy regimens, and who received either Grastofil^®^ or Neupogen^®^ as prophylaxis were included for analysis.

Patients were excluded if they received Grastofil^®^ or Neupogen^®^ after the start of the first cycle of chemotherapy (filgrastim was not given as prophylaxis), Grastofil^®^ or Neupogen^®^ was stopped while patients were still receiving treatment, they received previous chemotherapy (not chemo-naïve), they experienced previous FN, their chemotherapy regimen was changed, chemotherapy was stopped for reasons excluding FN, they were positive for HIV infection, or they were male breast cancer patients. Male breast cancer patients were excluded from this study due to difficulty in matching.

### 2.3. Data Collection

Research ethics board approval was obtained from our centre (REB# 224-2017). Our centre’s electronic medical record system was used to collect clinical data on patient, disease, and treatment characteristics, along with Grastofil^®^ and Neupogen^®^ usage. Clinical data were also cross-referenced with our centre’s oncology pharmacy management system.

Patient characteristics such as height and weight were collected to calculate body surface area (BSA) and planned dose intensity—baseline hemoglobin and age were collected for the purposes of matching. Sex (male or female) and bone marrow involvement were collected for matching lymphoma patients only. In addition, treatment characteristics, including chemotherapy regimen, date of each chemotherapy cycle, and administered doses for each chemotherapy agent were collected, along with the date and dose of Grastofil^®^ or Neupogen^®^ administered.

Across all cycles, the incidence of FN, DD, and DR associated with the use of G-CSF agent was evaluated. The general guidelines for FN diagnosis and standards of care directing G-CSF prophylaxis remained consistent throughout the duration of the study period.

### 2.4. Matching Criteria

Patients from Grastofil^®^ and Neupogen^®^ cohorts were case-matched and compared in a 1:1 manner on the following matching criteria: cancer site (breast cancer or non-Hodgkin’s lymphoma), chemotherapy regimen, age at the time of treatment (±5 years), baseline hemoglobin levels (<120 g/L vs. ≥120 g/L), sex (for lymphoma patients only) and bone marrow involvement (for lymphoma patients only). A propensity score (between 0 and 1) is the predicted probability of an outcome (e.g., treatment variable Z: 0 = Grastofil^®^ vs. 1 = Neupogen^®^) based on the subject’s observed covariates (e.g., vector of X). The propensity score *e*(*X*) for an individual is defined as the conditional probability of being treated with Neupogen^®^ given the subject’s covariates X: *e*(*X*) = Pr (Z = 1|*X*). The estimated propensity score $\hat{e}\left(X\right)$ is obtained from the fit of a logistic regression model for which we considered the above matching covariates. It has been shown that a sample matched on propensity score would be similar for all the covariates that went into computing the propensity score. Therefore, the propensity score is often used for matching case–control studies to reduce selection bias [27].

In the 1:1 matching, a set of A cases is matched to a set of B controls in a set of A decisions. To match subjects, we used an automated matching procedure in the SAS software that randomly selected a Neupogen^®^ individual and randomly selected a Grastofil^®^ individual (comparator) from the pool of potential comparators to determine whether the subject fulfilled the matching criteria. If the selected comparator was eligible, the subject was matched to the Neupogen^®^ individual, and the pair would not be reconsidered and was removed from the pool. This procedure was repeated until all Neupogen^®^ subjects were matched to one comparator or until no further comparators fulfilled the matching criteria. We used Statistical Analysis Software (SAS version 9.4, Cary, NC, USA) macro *OneToManyMTCH* with 8-digit to 1-digit match [27] and non-replacement to conduct this propensity score matching procedure. To evaluate the matched samples, a Wilcoxon rank-sum test or a Fisher exact test was used for comparing propensity scores and categorical matching criteria between two treatments.

### 2.5. Analysis

Demographics of patients included in the first and total cycle analysis were summarized using mean, standard deviation (SD), median, inter-quartiles, and range for continuous variables, and proportions for categorical variables. To compare demographics between Grastofil^®^ and Neupogen^®^ patients, a Wilcoxon rank-sum nonparametric test or a Fisher exact test was applied for continuous or categorical variables as appropriate. The primary objective of non-inferiority of the biosimilar vs. reference biologic was evaluated for the rate of FN in cycle 1 and for total cycles, where the risk difference (RD) in the rate of FN with 95% confidence intervals (CI) was reported. The non-inferiority margin was set at 15% for the absolute risk difference in the FN rate between treatments. Non-inferiority was met if the upper limit of 95% CI was <15%.

To compare FN incidence and the side effects of DD and DR between Grastofil^®^ and Neupogen^®^ treatment group in the whole cycles’ analysis, generalized estimating equation (GEE) models were conducted, and a binomial distribution with logit link function was specified in the GEE models. For the duration of FN-associated hospitalization in days and number of days delayed, a Poisson distribution with log link function was specified in the GEE model. All models were fit using an exchangeable correlation structure. The independent variables included the binary treatment group (Grastofil^®^ vs. Neupogen^®^) and chemotherapy cycles (1–8). *p*-value, RD, and 95% confidence intervals (CI) were estimated for each binary endpoint. All analyses were conducted using SAS, and a *p*-value <0.05 was considered statistically significant.

## 3. Results

### 3.1. Screening Results

**Figure 1:**
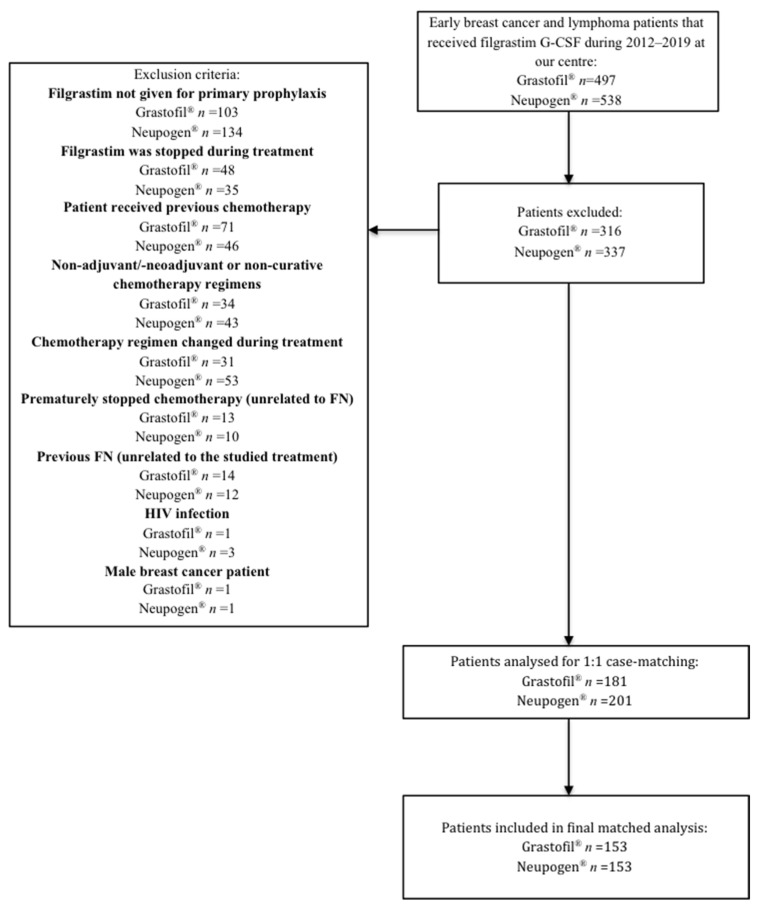
Exclusion criteria to determine cohorts.

A total of 497 breast cancer and lymphoma patients who received Grastofil^®^ were screened and 316 were excluded (Figure 1). Similarly, 538 early breast cancer and lymphoma patients who received Neupogen^®^ were screened and 336 were excluded.

### 3.2. Matching

Patients receiving Grastofil^®^ were matched in a 1:1 manner with a patient receiving Neupogen^®^. The matched analysis was completed using a propensity score weighting based on the characteristics mentioned in the Materials and Methods section. Among 181 eligible patients with Grastofil^®^, 153 patients had matched to 153 patients from the Neupogen^®^ group. Table 1 describes the baseline characteristics for these 153 patients from Grastofil^®^ and Neupogen^®^. There was no significant difference between the baseline characteristics of the cases and controls, indicating the well-matched pairs from Grastofil^®^ and Neupogen^®^ groups. The mean propensity scores were similar between the two groups (*p* = 0.99).

### 3.3. Patient Demographics

Patient demographics and treatment characteristics were summarized in breast cancer patients (Table 2) and in lymphoma patients (Table 3), respectively.

Among 275 patients in the breast site cohort (Table 2), there were 136 receiving Neupogen^®^ and 139 receiving Grastofil^®^. These represented a total of 1699 chemotherapy cycles, including 849 Neupogen^®^ cycles and 850 Grastofil^®^ cycles. There were no significant differences between the Grastofil^®^ and Neupogen^®^ patients in age categories, primary diagnosis, baseline hemoglobin levels, and chemotherapy regimen. There were significant differences in age (*p* = 0.04), disease stage (*p* = 0.01), and dose/number of doses (*p* < 0.0001) between Neupogen^®^ and Grastofil^®^ patients. Grastofil^®^ patients were more likely to be older (median 56 vs. 54 years), have lower proportions of disease stage ≥II (83% vs. 93%), and have higher mean dose/number of doses (40.3 vs. 36.7), as compared with patients on Neupogen^®^.

In the lymphoma cohort (Table 3), there were 31 patients, with 17 receiving Neupogen^®^ and 14 receiving Grastofil^®^. These represented a total of 162 chemotherapy cycles, including 89 Neupogen^®^ cycles and 73 Grastofil^®^ cycles, respectively. There were no significant differences between the Grastofil^®^ and Neupogen^®^ patients in age, sex, primary diagnosis, disease stage, baseline hemoglobin levels, and chemotherapy regimen. There was a significant difference in dose/number of doses (*p* = 0.04) between Neupogen^®^ and Grastofil^®^ patients (mean 33.6 vs. 34.1).

### 3.4. Incidence of Febrile Neutropenia in Breast Cancer Cohort

In the first cycle, six (4.32%) Grastofil^®^ patients and six (4.41%) Neupogen^®^ patients experienced an FN event, and the RD was −0.09% (95% CI: −4.92% to 4.73%), demonstrating non-inferiority of the biosimilar compared with the originator. Mean FN-associated hospitalization was also similar at 7.5 ± 1.05 days and 9.2 ± 4.40 days for Neupogen^®^ and Grastofil^®^ patients, respectively (*p* = 0.32) (Table 4).

In all eight combined cycles, there were 10 FN events (1.18%) in the Neupogen^®^ group and 13 FN events (1.53%) in the Grastofil^®^ group. After adjusting for chemotherapy cycles, the RD between the two groups (Grastofil^®^ vs. Neupogen^®^) was 0.3% (95% CI: −0.87% to 1.53%) for FN, demonstrating non-inferiority of the biosimilar compared with the originator. When comparing events between patients, there were 10 patients (7.4%) who experienced at least one FN event in the Neupogen^®^ group and 11 patients (7.9%) in the Grastofil^®^ group (RD = 0.5%; 95% CI −5.71 to 6.84%), demonstrating non-inferiority of the biosimilar compared with the originator (Table 5 and Table 6). Table A1 includes the cycle-per-cycle analysis of FN outcomes.

### 3.5. Incidence of Febrile Neutropenia in Lymphoma Cohort

The first cycle (Table 4) and all cycle events (Table 7 and Table 8) were separately analysed. In the first cycle, only one (5.88%) Neupogen^®^ patient and one (7.14%) Grastofil^®^ patient experienced an FN event (RD = 1.26%; 95% CI −16.26 to 18.79%). Since the upper level of 95% CI is >15%, this result does not demonstrate non-inferiority of the biosimilar compared with the originator. The duration of FN-associated hospitalization was also similar at 8 days and 7 days for one Neupogen^®^ and one Grastofil^®^ patient, respectively.

In all combined cycles (Table 7 and Table 8), there were four FN events (4.5%) in the Neupogen^®^ group and one (1.4%) in the Grastofil^®^ group. After adjusting for chemotherapy cycles, the RD was −3.1% (95% CI: −8.64% to 2.40%) for FN, demonstrating non-inferiority of the biosimilar compared with the originator. When comparing events between patients, there were three patients (17.7%) with at least one FN event in the Neupogen^®^ group and one patient (7.1%) in the Grastofil^®^ group (RD = −10.6%; 95% CI: −33.10% to 12.09%), demonstrating non-inferiority of the biosimilar compared with the originator. Table A2 includes the cycle-per-cycle analysis of FN outcomes.

### 3.6. Incidence of Dose Reductions and Dose Delays in Breast Cancer Cohort

From all 1699 chemotherapy cycles (Table 5 and Table 6), there were 27 dose-delayed cycles (3.18%) in the Neupogen^®^ group and 39 (4.59%) in the Grastofil^®^ group. After adjusting for chemotherapy cycles, the RD for DD was 1.4% (95% CI: −0.70% to 3.58%). When these data were correlated with the patient-level occurrence, there were 21 patients with at least one DD (15.4%) in the Neupogen^®^ group and 29 patients (20.9%) in the Grastofil^®^ group (RD = 5.5%; 95% CI: −3.66% to 14.51%).

There were 166 cycles with a DR (19.6%) in the Neupogen^®^ group and 211 cycles (24.8%) in the Grastofil^®^ group. The RD for DR was 5.2% (95% CI: −0.23% to 11.31%) after adjusting for chemotherapy cycles. There were 73 (53.7%) patients who experienced at least one cycle with a DR in the Neupogen^®^ group and 79 patients (56.8%) in the Grastofil^®^ group (RD = 3.1%; 95% CI −8.59 to 14.91%). Table A1 includes the cycle-per-cycle analysis of DR and DD.

### 3.7. Incidence of Dose Reductions and Dose Delays in Lymphoma Cohort

From all 162 cycles (Table 7 and Table 8), there were 4/89 cycles (4.5%) that experienced a DD in the Neupogen^®^ group and 1/73 cycles (1.4%) in the Grastofil^®^ group (RD = −3.1%; 95% CI: −8.64% to 2.40% after adjusting for chemotherapy cycles). There were 2/17 patients (11.8%) who experienced at least one DD cycle in the Neupogen^®^ group and 1/14 patients (7.1%) in the Grastofil^®^ group (RD = −4.7%; 95% CI: −25.03% to 15.79%).

There were 14/89 cycles (15.7%) with a DR in the Neupogen^®^ group and 10/73 reduced cycles (13.7%) in the Grastofil^®^ group (RD = −2.0%; 95% CI: −21.20% to 15.28% after adjusting for chemotherapy cycles). When looking at the patient-level data, there were 4/17 (23.5%) patients with at least one DR in the Neupogen^®^ group and 4/14 patients (28.6%) in the Grastofil^®^ group (RD = 5.1%; 95% CI: −26.05% to 36.13%). Table A2 includes the cycle-per-cycle analysis of DR and DD.

## 4. Discussion

This study determined non-inferiority of the biosimilar Grastofil^®^ to the reference product Neupogen^®^ for the prophylaxis of FN in early breast cancer and lymphoma patients at our centre. Our study’s findings further support clinical data and the recommendation put forth by the CADTH CDEC [28]. This is also in alignment with a 2018 meta-analysis by Botteri et al. that compared the safety and efficacy of the pegfilgrastim and filgrastim biologics to their respective biosimilars, as was reported in the randomized controlled trials with breast cancer patients [21]. In the eight RCTs included, there was no significant difference in both duration of FN (mean difference = 0.06 days; 95% CI −0.05 to 0.17) and overall occurrence of FN (RR = 0.96; 95% CI 0.71–1.3) between either the long- and short-acting GCSFs vs. their respective biosimilars [21].

The overall first cycle FN rate, including both breast cancer and lymphoma cohorts, was 7/153 (4.58%) in both the Neupogen^®^ and Grastofil^®^ cohorts. In the breast cancer cohort (*n* = 275), the first cycle FN rate was generally higher than in subsequent cycles, as 6 among a total of 10 FN events occurred in the first cycle for Neupogen^®^, and 6 among a total of 13 FN events occurred in the first cycle for Grastofil^®^. This aligns with previous literature, as the first cycle has been previously cited to be the highest risk for a neutropenic event [29,30,31]. A 2008 nationwide prospective study by Crawford et al. studied 2962 cancer patients, where 58.9% of their observed neutropenic events occurred in the first cycle [31].

Furthermore, the FN incidence of this sample aligns closely with another retrospective study conducted from our previous analysis of pegfilgrastim biosimilar clinical comparability [32]. This study by Wong et al. compared the pegfilgrastim biosimilar to its reference biologic in breast cancer patients (*n* = 174) and reported an overall first cycle FN rate of 3.45%, with 5.41% from the biosimilar and 2% in the reference biologic [32]. Our results show similar FN rates of 4.4% in the Neupogen^®^ group and 4.3% in the Grastofil^®^ group. Similarly, the trend of higher FN incidence in the first cycle was reported in this study, with over 60% of all reported FN events occurring in the first cycle [32]. The results of this study also support the non-inferiority of the biosimilar product. The consistency in these results further supports the feasibility of the sample used in the current study.

This study included a small subset of lymphoma patients who received chemotherapy at our centre. The inclusion of both solid and haematological cancers reflected a real-world perspective for the usage of this G-CSF product amongst more complex populations and generalizability of findings. The analysis included in this study separated the lymphoma and breast cancer patients, as these two distinct groups have very different clinical features. Firstly, the myelosuppressive treatments received are quite distinct and may not be equally comparable between the two cohorts. Additionally, there are more men in the lymphoma population than in the breast cancer population. Since there have been observed differences in the risk of FN in men vs. women (incidence risk ratio = 1.39; 95% CI = 1.06–1.81), separating the breast cancer group from the lymphoma group, would decrease inconsistency in the results [33]. Additionally, a retrospective study by Lyman et al. evaluated risk factors for FN prevalence in 4091 patients receiving myelosuppressive chemotherapy [34]. When non-Hodgkin’s lymphoma was compared with breast cancer, there was a slightly decreased OR trend in breast cancer, though this difference was non-significant (OR = 0.92; 95% CI: 0.65–1.30, *p* = 0.6) [34]. This demonstrates that there may be potential differences in the prognosis and risk of FN development [34].

Notably, the first cycle FN outcomes could not demonstrate non-inferiority in the lymphoma cohort, but in the whole cycle analysis, non-inferiority was shown. This may be attributed to the higher prevalence of patients who presented with FN in the Grastofil^®^ cohort. However, the overall FN rates equalized, as more Neupogen^®^ patients experienced FN in cycles 4 and 5.

The secondary objective included the comparison of DD and DR, as these are important in assessing the chemotherapy treatment outcomes associated with decreased morbidity or mortality. A study by Veitch et al. consisted of 1302 breast cancer patients, investigating the relationship between DD, DR, and survival, and reported that patients who received ≥85% of the prescribed dose had better 5-year disease-free survival than those who received <85% of the prescribed dose (85.9% vs. 79.2%; *p* = 0.025) while also showing superior overall survival at 5 years (88.8% vs. 80.7%; *p* < 0.001) [35]. Furthermore, they found that patients who received the dose reduction >15% in earlier cycles were associated with poorer overall survival than a dose reduction in a later cycle (HR = 1.77; 95% CI: 1.14–2.75, *p* = 0.1) [35]. They also reported no significant association with dose delay and disease-free survival or overall survival [35]. In a retrospective study in 3866 lung cancer patients, Crawford et al. found similar results, where 32.4% of patients experienced a DD, and 50.1% experienced a DR. Furthermore, they found that relative dose intensity ≥85% was associated with an 18% decrease in risk of death [36].

At the time of submission, Grastofil^®^ (CAD 144.31 per 300 mcg/0.5 mL pre-filled syringe) was 25% less costly than Neupogen^®^ (CAD 192.42 per 300 mcg/mL vial) for all Health Canada approved indications [28]. Currently, Grastofil^®^ is listed on the ODB formulary at the same submitted price for the 300 mcg/0.5 mL pre-filled syringe and CAD 230.90 per 480 mcg/mL pre-filled syringe, compared with Neupogen^®^, which is now listed on ODB limited use for CAD 176.13 per 300 mcg/mL vial and CAD 281.81 per 480 mcg/1.6 mL vial [22]. Grastofil^®^ remains less costly than Neupogen^®^ at current listed prices, at approximately 17% less for both 300 mcg and 480 mcg doses.

Multiple studies support the cost savings and increased access to drug products imparted through the use of biosimilars, thereby increasing the value of cancer care delivered [17,37,38,39]. However, there is still skepticism towards the widespread utilization of these products. A 2016 US-based study evaluated responses to a 19-question survey from 1201 specialists regarding their perceptions, comfort, and awareness of the use of biosimilars [40]. When asked whether specialists believed if biosimilars were safe and appropriate for use in naïve patients, over 30% of specialists believed that biosimilars were less safe than the originator due to an abbreviated pathway of approval [40]. Therefore, in this growing field, research studies are particularly important. Our study, together with continued research following future biosimilar drug approvals on their real-world effectiveness, aims to reduce any persisting stigmatization associated with their use and to encourage continued physician adoption.

A strength of this study was its ability to view the clinical comparability of the drug of interest in a non-trial specific setting. Often in phase III trials, the study population has strict inclusion and exclusion criteria and often underrepresents important subgroup populations. This retrospective study did not discriminate based on pre-determined demographics, thus providing a more robust comparison of this drug product in a relatively large sample size. Furthermore, the 1:1 matched propensity score analysis allowed for the two comparison groups to be as similar as possible and decreased the potential confounding impact of various other treatment-related factors. However, a limitation of this study is related to its retrospective nature, as the reliance on electronic health records can mean certain events may be underreported. This study was a retrospective review, and therefore, the collection of some clinical data, such as information regarding the reason for DD and DR, was not reported. Consequently, it is not apparent whether every DD or DR was associated with a neutropenic event plus the majority of reports on FN were limited to instances recorded at our centre, and therefore, there is a possibility that visits to other centres for FN were not accounted for in this study. Furthermore, another potential limitation is the utility of a historical cohort, as this may not be completely representative of the current treatments. However, since the collection of these data, there have not been many extensive changes to our institutional standard of care. In the event of significant protocol changes, the study would still provide useful information about the clinical comparability of these two supportive care drugs at a given point in time. Therefore, the results of this study still effectively capture the safety and efficacy of biosimilars. Overall, this was consistent throughout the data collection process for both Grastofil^®^ and Neupogen^®^ cohorts.

## 5. Conclusions

The findings of this study support previously documented literature on the clinical comparability of biosimilars. This retrospective assessment of FN incidence in breast cancer and lymphoma patients receiving biosimilar Grastofil^®^ was found to be statistically non-inferior to rates observed with the use of Neupogen^®^. Furthermore, the rates of DD and DR in the biosimilar Grastofil^®^ were also non-inferior to the reference Neupogen^®^ product. In a real-world setting, the filgrastim biosimilar Grastofil^®^ has demonstrated comparable safety and efficacy with the originator. Therefore, increasing its adoption in myelosuppressive cancer patients can result in decreased health expenditure and increased patient choice, all while providing a similar level of care.

## Figures and Tables

**Table 1:** Propensity Score Matching.

Matched Category	Treatment		*p*-Value
Baseline and Characteristics	Neupogen^®^ (*N* = 153)	Grastofil^®^ (*N* = 153)	
Age (years)			0.3895
<40	20 (13.07%)	12 (7.84%)	
40 to < 50	37 (24.18%)	37 (24.18%)	
50 to < 60	32 (20.92%)	29 (18.95%)	
≥60	64 (41.83%)	75 (49.02%)	
Cancer site			0.7053
Breast	136 (88.89%)	139 (90.85%)	
Hematology	17 (11.11%)	14 (9.15%)	
Chemo regimen			0.6349
AC-PACL	56 (36.60%)	56 (36.60%)	
CHOMP + RITUX	3 (1.96%)	1 (0.65%)	
CHOP	13 (8.50%)	13 (8.50%)	
Docetaxcyclo	24 (15.69%)	24 (15.69%)	
EPOCH-RITUX	1 (0.65%)	0 (0.00%)	
FEC-D	56 (36.60%)	56 (36.60%)	
TCH	0 (0.00%)	3 (1.96%)	
Baseline HgB < 120			0.5109
No	129 (84.31%)	134 (87.58%)	
Yes	24 (15.69%)	19 (12.42%)	
Propensity Score (Mean ± SD)	0.5255 ± 0.0982	0.5255 ± 0.0982	0.9999

AC-PACL = doxorubicin, cyclophosphamide, paclitaxel; CHOMP+RITUX = cyclophosphamide, doxorubicin, vincristine, prednisone, methotrexate, and rituximab; CHOP = prednisone, doxorubicin, vincristine, cyclophosphamide; DOCETAXCYCLO = docetaxel, cyclophosphamide; EPOCH-RITUX = etoposide, vincristine, doxorubicin, prednisone, cyclophosphamide, rituximab; FEC-D = fluorouracil, epirubicin, cyclophosphamide, docetaxel; TCH = docetaxel, carboplatin, trastuzumab.

**Table 2:** Demographics in Breast Cancer Patients Only.

Demographics	Total (*N* = 275)	Neupogen^®^ (*N* = 136)	Grastofil^®^ (*N* = 139)	*p*-Value *
**Age (years)**				**0.0403**
*N*	275	136	139	
Mean ± SD	54.70 ± 12.30	53.07 ± 12.36	56.30 ± 12.08	
Median (Inter-quartiles)	55.0 (45.0, 66.0)	54.0 (44.0, 63.0)	56.0 (46.0, 67.0)	
Min, Max	27.0, 77.0	27.0, 76.0	27.0, 77.0	
Age (years)				0.1034
<40	30 (10.91%)	20 (14.71%)	10 (7.19%)	
40 to <50	74 (26.91%)	37 (27.21%)	37 (26.62%)	
50 to <60	60 (21.82%)	32 (23.53%)	28 (20.14%)	
≥60	111 (40.36%)	47 (34.56%)	64 (46.04%)	
Sex				NA
Female	275 (100.00%)	136 (100.00%)	139 (100.00%)	
Male	0 (0.00%)	0 (0.00%)	0 (0.00%)	
Primary diagnosis				0.3505
DCIS	2 (0.73%)	0 (0.00%)	2 (1.44%)	
IDC	247 (89.82%)	125 (91.91%)	122 (87.77%)	
IDC/ILC	4 (1.45%)	2 (1.47%)	2 (1.44%)	
ILC	16 (5.82%)	8 (5.88%)	8 (5.76%)	
IMC	6 (2.18%)	1 (0.74%)	5 (3.60%)	
Hemoglobin at baseline (g/L)				0.5755
*N*	275	136	139	
Mean ± SD	133.09 ± 10.31	133.28 ± 10.17	132.91 ± 10.47	
Median (Inter-quartiles)	134.0 (128.0, 140.0)	134.0 (128.5, 140.0)	133.0 (127.0, 140.0)	
Min, Max	102.0, 157.0	105.0, 154.0	102.0, 157.0	
Hemoglobin at baseline < 120 (g/L)				0.7093
No	243 (88.36%)	119 (87.50%)	124 (89.21%)	
Yes	32 (11.64%)	17 (12.50%)	15 (10.79%)	
**Disease Stage ≥ II at baseline**				**0.0102**
No	14 (5.09%)	6 (4.41%)	8 (5.76%)	
Yes	243 (88.36%)	127 (93.38%)	116 (83.45%)	
Not available	18 (6.55%)	3 (2.21%)	15 (10.79%)	
Chemo regimens				0.5022
AC-PACL	112 (40.73%)	56 (41.18%)	56 (40.29%)	
Docetaxcyclo	48 (17.45%)	24 (17.65%)	24 (17.27%)	
FEC-D	112 (40.73%)	56 (41.18%)	56 (40.29%)	
TCH	3 (1.09%)	0 (0.00%)	3 (2.16%)	
**Dose (mcg)/number of doses**				**<0.0001**
*N*	275	136	139	
Mean ± SD	38.51 ± 7.60	36.72 ± 7.40	40.25 ± 7.40	
Median (Inter-quartiles)	37.5 (37.5, 37.5)	37.5 (30.0, 37.5)	37.5 (37.5, 37.5)	
Min, Max	30.0, 80.0	30.0, 80.0	37.5, 60.0	

* *p*-value was obtained by Wilcoxon rank-sum nonparametric test or Fisher exact for continuous or categorical variables as appropriate. *p* < 0.05 was considered statistically significant (Bolded). DCIS= ductal carcinoma in situ; IDC= invasive ductal carcinoma; ILC= invasive lobular carcinoma; IMC= invasive mammary carcinoma.

**Table 3:** Demographics in Lymphoma Patients Only.

Demographics	Total (*N* = 31)	Neupogen^®^ (*N* = 17)	Grastofil^®^ (*N* = 14)	*p*-Value *
Age (years)				0.8271
N	31	17	14	
Mean ± SD	71.84 ± 13.69	74.71 ± 8.04	68.36 ± 18.14	
Median (Inter-quartiles)	75.0 (66.0, 81.0)	73.0 (70.0, 81.0)	75.0 (66.0, 80.0)	
Min, Max	27.0, 90.0	61.0, 90.0	27.0, 85.0	
Age (years)				0.0810
<40	2 (6.45%)	0 (0.00%)	2 (14.29%)	
40 to <50	0 (0.00%)	0 (0.00%)	0 (0.00%)	
50 to <60	1 (3.23%)	0 (0.00%)	1 (7.14%)	
≥60	28 (90.32%)	17 (100.00%)	11 (78.57%)	
Sex				0.7087
Female	21 (67.74%)	12 (70.59%)	9 (64.29%)	
Male	10 (32.26%)	5 (29.41%)	5 (35.71%)	
Primary diagnosis				0.8487
ATLL ^1^	1 (3.23%)	1 (5.88%)	0 (0.00%)	
DLBCL ^2^	28 (90.32%)	15 (88.24%)	13 (92.86%)	
HGBL ^3^	1 (3.23%)	1 (5.88%)	0 (0.00%)	
TFL ^4^	1 (3.23%)	0 (0.00%)	1 (7.14%)	
Hemoglobin at baseline (g/L)				0.9842
N	31	17	14	
Mean ± SD	122.32 ± 22.74	120.00 ± 22.86	125.14 ± 23.11	
Min, Max	71.0, 173.0	79.0, 146.0	71.0, 173.0	
Hemoglobin at baseline < 120 (g/L)				0.7074
No	20 (64.52%)	10 (58.82%)	10 (71.43%)	
Yes	11 (35.48%)	7 (41.18%)	4 (28.57%)	
Disease Stage ≥ II at baseline				0.0990
No	5 (16.13%)	1 (5.88%)	4 (28.57%)	
Yes	24 (77.42%)	14 (82.35%)	10 (71.43%)	
Not available	2 (6.45%)	2 (11.76%)	0 (0.00%)	
Chemo regimens				0.6067
CHOMP + RITUX ^5^	4 (12.90%)	3 (17.65%)	1 (7.14%)	
CHOP ^6^	26 (83.87%)	13 (76.47%)	13 (92.86%)	
EPOCH-RITUX ^7^	1 (3.23%)	1 (5.88%)	0 (0.00%)	
**Dose (mcg)/number of doses**				**0.0404**
N	31	17	14	
Mean ± SD	33.81 ± 6.41	33.62 ± 7.10	34.05 ± 5.73	
Median (Inter-quartiles)	30.0 (30.0, 33.3)	30.0 (30.0, 30.0)	33.3 (33.3, 33.3)	
Min, Max	30.0, 53.3	30.0, 48.0	30.0, 53.3	

* *p*-value was obtained by Wilcoxon rank-sum nonparametric test or Fisher exact for continuous or categorical variables as appropriate. *p* < 0.05 was considered statistically significant (Bolded). ^1^ ATLL = adult T-cell leukemia/lymphoma; ^2^ DLBCL = diffuse large B-cell lymphoma; ^3^ HGBL = high-grade B-cell lymphoma; ^4^ TFL = transformed follicular lymphoma; ^5^ CHOMP + RITUX = cyclophosphamide, doxorubicin, vincristine, prednisone, methotrexate, and rituximab; ^6^ CHOP = prednisone, doxorubicin, vincristine, cyclophosphamide; ^7^ EPOCH-RITUX = etoposide, vincristine, doxorubicin, prednisone, cyclophosphamide, rituximab.

**Table 4:** First Cycle Febrile Neutropenia Outcomes.

Breast Cancer Patients	Total (*N* = 275)	Neupogen^®^ (*N* = 136)	Grastofil^®^ (*N* = 139)	*p*-Value	RD *	95% CI of RD
Febrile Neutropenia (FN)				0.9692	−0.09%	−4.92 to 4.73%
No	263 (96.03%)	130 (95.59%)	133 (95.68%)			
Yes	12(4.36%)	6 (4.41%)	6 (4.32%)			
FN-Associated Hospitalization (days)				0.3181	N/A	N/A
N	12	6	6			
Mean ± SD	8.33 ± 3.17	7.50 ± 1.05	9.17 ± 4.40			
Min, Max	6.0, 18.0	6.0, 9.0	6.0, 18.0			
**Lymphoma Patients**	**Total (*N* = 31)**	**Neupogen^®^ (*N* = 17)**	**Grastofil^®^ (*N* = 14)**	***p*-Value**	RD *	**95% CI of RD**
Febrile Neutropenia (FN)				0.8871	1.26%	−16.26 to 18.79%
No	29 (93.55%)	16 (94.12%)	13 (92.86%)			
Yes	2 (6.45%)	1 (5.88%)	1 (7.14%)			
FN-Associated Hospitalization (days)				0.7964	N/A	N/A
N	2	1	1			
Mean ± SD	7.50 ± 0.71	8.00 ± NA	7.00 ± NA			
Min, Max	7.0, 8.0	8.0, 8.0	7.0, 7.0			

* *p*-value, risk difference (RD) and 95% confidence interval (CI) were calculated using generalized estimating equation (GEE) model with a binomial distribution and logit link function.

**Table 5:** Combined Cycle Breast Cancer Outcomes.

Combined ALL Cycles	Total *N* = 1699	Neupogen^®^ *N* = 849	Grastofil^®^ *N* = 850	*p*-Value *
Febrile Neutropenia (FN)				0.6755
No	1676 (98.65%)	839 (98.82%)	837 (98.47%)	
Yes	23 (1.35%)	10 (1.18%)	13 (1.53%)	
FN-Associated Hospitalization (days)				0.7018
N	23	10	13	
Mean ± SD	8.0 ± 2.6	7.9 ± 1.7	8.2 ± 3.2	
Median (Inter-quartiles)	8.0 (6.0, 8.0)	8.0 (7.0, 8.0)	7.0 (6.0, 8.0)	
Min, Max	6, 18	6, 12	6, 18	
Dose delays				0.1668
No	1633 (96.12%)	822 (96.82%)	811 (95.41%)	
Yes	66 (3.88%)	27 (3.18%)	39 (4.59%)	
No. of days delayed				0.7026
N	66	27	39	
Mean ± SD	9.5 ± 5.5	9.1 ± 4.2	9.8 ± 6.3	
Median (Inter-quartiles)	7.0 (7.0, 8.0)	7.0 (7.0, 8.0)	7.0 (7.0, 8.0)	
Min, Max	6, 35	6, 21	6, 35	
**Dose reduction**				**0.0101**
No	1322 (77.81%)	683 (80.45%)	639 (75.18%)	
Yes	377 (22.19%)	166 (19.55%)	211 (24.82%)	
**Total Outcomes per Patient**	**Total** ***N* = 275**	**Neupogen^®^** ***N* = 136**	**Grastofil^®^** ***N* = 139**	*p*-Value *
Febrile Neutropenia (FN)				0.8610
No	254 (92.36%)	126 (92.65%)	128 (92.09%)	
Yes	21 (7.64%)	10 (7.35%)	11 (7.91%)	
FN-Associated Hospitalization (days)				0.7189
*N*	21	10	11	
Mean ± SD	8.8 ± 3.7	7.9 ± 1.7	9.6 ± 4.8	
Median (Inter-quartiles)	8.0 (7.0, 9.0)	8.0 (7.0, 8.0)	8.0 (7.0, 10.0)	
Min, Max	6, 20	6, 12	6, 20	
Dose delays				0.2754
No	225 (81.82%)	115 (84.56%)	110 (79.14%)	
Yes	50 (18.18%)	21 (15.44%)	29 (20.86%)	
Total no. of days delayed				0.9011
*N*	50	21	29	
Mean ± SD	12.6 ± 8.9	11.7 ± 7.3	13.2 ± 10.0	
Median (Inter-quartiles)	7.0 (7.0, 17.0)	7.0 (7.0, 17.0)	7.0 (7.0, 14.0)	
Min, Max	6, 49	6, 28	6, 49	
Dose reduction				0.6287
No	123 (44.73%)	63 (46.32%)	60 (43.17%)	
Yes	152 (55.27%)	73 (53.68%)	79 (56.83%)	

* *p*-value was obtained by Wilcoxon rank-sum nonparametric test or Fisher exact for continuous or categorical variables as appropriate. *p* < 0.05 was considered statistically significant (Bolded).

**Table 6:** Combined Cycle Breast Cancer Risk Differences.

Comparing Each of Endpoints between Grastofil^®^ and Neupogen^®^ in Total Analysis, after Adjusting for Chemotherapy Cycles			
Endpoints	*p*-Value **	RD (95% CI of RD) between Grastofil^®^ and Neupogen^®^	
FN (Yes vs. No)	0.7047	0.3%	(−0.87 to 1.53%)
FN-Associated Hospitalization (days)	0.5625	N/A	N/A
Dose delayed (Yes vs. No)	0.1761	1.4%	(−0.7 to 3.58%)
Number of days of dose delayed	0.6723	N/A	N/A
**Dose reductions (Yes vs. No)**	**0.0438**	**5.2%**	**(−0.23 to 11.31%)**

** *p*-value was obtained by GEE model for this longitudinal data in combined all cycles’ analysis, after adjusting for chemotherapy cycles. RD and 95% CI were also calculated by GEE model.

**Table 7:** Combined Lymphoma Outcomes.

Outcomes	Total	Neupogen^®^	Grastofil^®^	
Combined All Cycles Outcomes	*N* = 162	*N* = 89	*N* = 73	*p*-Value *
Febrile Neutropenia (FN)				0.3796
No	157 (96.91%)	85 (95.51%)	72 (98.63%)	
Yes	5 (3.09%)	4 (4.49%)	1 (1.37%)	
FN-Associated Hospitalization (days)				0.2888
*N*	5	4	1	
Mean ± SD	11.6 ± 4.2	12.8 ± 3.8	7.0 ± NA	
Min, Max	7, 17	8, 17	7, 7	
Dose delayed				0.3796
No	157 (96.91%)	85 (95.51%)	72 (98.63%)	
Yes	5 (3.09%)	4 (4.49%)	1 (1.37%)	
No. of days of dose delayed				0.2765
*N*	5	4	1	
Mean ± SD	12.0 ± 6.6	9.8 ± 4.9	21.0 ± NA	
Min, Max	7, 21	7, 17	21, 21	
Dose reduction				0.8254
No	138 (85.19%)	75 (84.27%)	63 (86.30%)	
Yes	24 (14.81%)	14 (15.73%)	10 (13.70%)	
**Total Outcomes per Patient**	***N*** **= 31**	***N*** **= 17**	***N*** **= 14**	***p*** -Value *
Febrile Neutropenia (FN)				0.6067
No	27 (87.10%)	14 (82.35%)	13 (92.86%)	
Yes	4 (12.90%)	3 (17.65%)	1 (7.14%)	
FN-Associated Hospitalization (days)				0.3711
*N*	4	3	1	
Mean ± SD	14.5 ± 5.6	17.0 ± 3.0	7.0 ± NA	
Min, Max	7, 20	14, 20	7, 7	
Dose delayed				0.6649
No	28 (90.32%)	15 (88.24%)	13 (92.86%)	
Yes	3 (9.68%)	2 (11.76%)	1 (7.14%)	
Total no. of days of dose delayed				0.9999
*N*	3	2	1	
Mean ± SD	20.0 ± 12.5	19.5 ± 17.7	21.0 ± NA	
Min, Max	7, 32	7, 32	21, 21	
Dose reduction				0.7495
No	23 (74.19%)	13 (76.47%)	10 (71.43%)	
Yes	8 (25.81%)	4 (23.53%)	4 (28.57%)	
				

* *p*-value was obtained by Wilcoxon rank-sum nonparametric test or Fisher exact for continuous or categorical variables as appropriate.

**Table 8:** Combined Lymphoma Risk Differences.

Comparing Each of Endpoints between Grastofil^®^ and Neupogen^®^ in Total Analysis, after Adjusting for Chemotherapy Cycles			
Endpoints	*p*-Value **	RD (95% CI) between Grastofil^®^ and Neupogen^®^	
FN (Yes vs. No)	0.2902	−3.12%	(−8.64 to 2.40%)
FN-Associated Hospitalization (days)	**<0.0001**	N/A	N/A
Dose delayed (Yes vs. No)	0.2506	−3.12%	(−8.64 to 2.40%)
Number of days of dose delayed	**<0.0001**	N/A	N/A
Dose reductions (Yes vs. No)	0.8802	−2.03%	(−21.20 to 15.28%)

** *p*-value was obtained by GEE model for this longitudinal data in combined all cycles’ analysis, after adjusting for chemotherapy cycles. RD and 95% CI were also calculated by GEE model. *p* < 0.05 was considered statistically significant (Bolded).

## Data Availability

The data used in this study are available upon request from the corresponding author.

## Appendix A

**Table A1:** Cycle-per-Cycle Outcomes in Breast Cancer Patients.

Per Cycle Analysis	Total	Neupogen^®^	Grastofil^®^	*p*-Value *
**At Cycle 1**	***N*** **= 275**	***N*** **= 136**	***N*** **= 139**	
Febrile Neutropenia (FN)				0.9692
No	263 (95.64%)	130 (95.59%)	133 (95.68%)	
Yes	12 (4.36%)	6 (4.41%)	6 (4.32%)	
FN-Associated Hospitalization (days)				0.7374
*N*	12	6	6	
Mean ± SD	8.33 ± 3.17	7.50 ± 1.05	9.17 ± 4.40	
Median (Inter-quartiles)	8.0 (7.0, 8.0)	7.5 (7.0, 8.0)	8.0 (7.0, 8.0)	
Min, Max	6.0, 18.0	6.0, 9.0	6.0, 18.0	
**At Cycle 2**	***N*** **= 275**	***N*** **= 136**	***N*** **= 139**	
Febrile Neutropenia (FN)				0.9876
No	273 (99.27%)	135 (99.26%)	138 (99.28%)	
Yes	2 (0.73%)	1 (0.74%)	1 (0.72%)	
FN-Associated Hospitalization (days)				NA
*N*	2	1	1	
Mean ± SD	9.0 ± 1.4	8.0 ± NA	10.0 ± NA	
Median (Inter-quartiles)	9 (8, 10)	8 (8, 8)	10 (10, 10)	
Min, Max	8, 10	8, 8	10, 10	
Dose delays				0.9692
No	263 (95.64%)	130 (95.59%)	133 (95.68%)	
Yes	12 (4.36%)	6 (4.41%)	6 (4.32%)	
No. of days delayed				0.8489
*N*	12	6	6	
Mean ± SD	8.9 ± 4.9	7.2 ± 0.4	10.7 ± 6.7	
Median (Inter-quartiles)	7 (7, 8)	7 (7, 7)	7 (7, 14)	
Min, Max	6, 23	7, 8	6, 23	
Dose reduction				0.1792
No	219 (79.64%)	113 (83.09%)	106 (76.26%)	
Yes	56 (20.36%)	23 (16.91%)	33 (23.74%)	
**At Cycle 3**	***N*** **= 273**	***N*** **= 134**	***N*** **= 139**	
Febrile Neutropenia (FN)				0.4908
No	272 (99.63%)	133 (99.25%)	139 (100.00%)	
Yes	1 (0.37%)	1 (0.75%)	0 (0.00%)	
FN-Associated Hospitalization (days)				NA
*N*	1	1	0	
Mean ± SD	12.0 ± NA	12.0 ± NA	-	
Median (Inter-quartiles)	12 (12, 12)	12 (12, 12)	-	
Min, Max	12, 12	12, 12	-	
Dose delays				0.7229
No	265 (97.07%)	131 (97.76%)	134 (96.40%)	
Yes	8 (2.93%)	3 (2.24%)	5 (3.60%)	
No. of days delayed				0.1685
*N*	8	3	5	
Mean ± SD	8.1 ± 2.4	7.0 ± 0.0	8.8 ± 2.9	
Median (Inter-quartiles)	7 (7, 8)	7 (7, 7)	8 (7, 8)	
Min, Max	7, 14	7, 7	7, 14	
Dose reduction				0.6734
No	206 (75.46%)	103 (76.87%)	103 (74.10%)	
Yes	67 (24.54%)	31 (23.13%)	36 (25.90%)	
**At Cycle 4**	***N*** **= 268**	***N*** **= 133**	***N*** **= 135**	
Febrile Neutropenia (FN)				0.6840
No	262 (97.76%)	131 (98.50%)	131 (97.04%)	
Yes	6 (2.24%)	2 (1.50%)	4 (2.96%)	
FN-Associated Hospitalization (days)				0.9999
*N*	6	2	4	
Mean ± SD	7.0 ± 1.3	7.0 ± 1.4	7.0 ± 1.4	
Median (Inter-quartiles)	7 (6, 8)	7 (6, 8)	7 (6, 8)	
Min, Max	6, 9	6, 8	6, 9	
Dose delays				0.7974
No	255 (95.15%)	127 (95.49%)	128 (94.81%)	
Yes	13 (4.85%)	6 (4.51%)	7 (5.19%)	
No. of days delayed				0.4385
*N*	13	6	7	
Mean ± SD	8.2 ± 3.6	9.3 ± 5.2	7.1 ± 0.4	
Median (Inter-quartiles)	7 (7, 7)	7 (7, 8)	7 (7, 7)	
Min, Max	7, 20	7, 20	7, 8	
Dose reduction				0.9201
No	219 (81.72%)	109 (81.95%)	110 (81.48%)	
Yes	49 (18.28%)	24 (18.05%)	25 (18.52%)	
**At Cycle 5**	***N*** **= 215**	***N*** **= 110**	***N*** **= 105**	
Febrile Neutropenia (FN)				NA
No	215 (100.00%)	110 (100.00%)	105 (100.00%)	
Yes	0 (0.00%)	0 (0.00%)	0 (0.00%)	
FN-Associated Hospitalization (days)				NA
*N*	0	0	0	
Mean ± SD	-	-	-	
Median (Inter-quartiles)	-	-	-	
Min, Max	-	-	-	
Dose delays				0.2440
No	203 (94.42%)	106 (96.36%)	97 (92.38%)	
Yes	12 (5.58%)	4 (3.64%)	8 (7.62%)	
No. of days delayed				0.5074
N	12	4	8	
Mean ± SD	11.3 ± 7.2	12.3 ± 6.7	10.8 ± 7.8	
Median (Inter-quartiles)	7 (7, 16)	11 (7, 18)	7 (7, 12)	
Min, Max	6, 28	7, 21	6, 28	
Dose reduction				0.0920
No	172 (80.00%)	93 (84.55%)	79 (75.24%)	
Yes	43 (20.00%)	17 (15.45%)	26 (24.76%)	
**At Cycle 6**	***N*** **= 209**	***N*** **= 107**	***N*** **= 102**	
Febrile Neutropenia (FN)				0.2370
No	207 (99.04%)	107 (100.00%)	100 (98.04%)	
Yes	2 (0.96%)	0 (0.00%)	2 (1.96%)	
FN duration (days)				NA
*N*	2	0	2	
Mean ± SD	6.5 ± 0.7	-	6.5 ± 0.7	
Median (Inter-quartiles)	7 (6, 7)	-	7 (6, 7)	
Min, Max	6, 7	-	6, 7	
Dose delays				0.2434
No	197 (94.26%)	103 (96.26%)	94 (92.16%)	
Yes	12 (5.74%)	4 (3.74%)	8 (7.84%)	
No. of days of dose delayed				0.3187
*N*	12	4	8	
Mean ± SD	9.6 ± 3.8	11.8 ± 5.1	8.5 ± 2.8	
Median (Inter-quartiles)	7 (7, 13)	12 (8, 16)	7 (7, 10)	
Min, Max	6, 17	6, 17	7, 14	
**Dose reduction**				**0.0100**
No	130 (62.20%)	76 (71.03%)	54 (52.94%)	
Yes	79 (37.80%)	31 (28.97%)	48 (47.06%)	
**At Cycle 7**	***N*** **= 96**	***N*** **= 49**	***N*** **= 47**	
Febrile Neutropenia (FN)				NA
No	96 (100.00%)	49 (100.00%)	47 (100.00%)	
Yes	0 (0.00%)	0 (0.00%)	0 (0.00%)	
FN-Associated Hospitalization (days)				NA
*N*	0	0	0	
Mean ± SD	-	-	-	
Median (Inter-quartiles)	-	-	-	
Min, Max	-	-	-	
Dose delays				0.4307
No	90 (93.75%)	47 (95.92%)	43 (91.49%)	
Yes	6 (6.25%)	2 (4.08%)	4 (8.51%)	
No. of days delayed				0.8057
*N*	6	2	4	
Mean ± SD	13.0 ± 11.1	7.5 ± 0.7	15.8 ± 13.3	
Median (Inter-quartiles)	8 (7, 14)	8 (7, 8)	11 (7, 25)	
Min, Max	7, 35	7, 8	7, 35	
Dose reduction				0.8630
No	56 (58.33%)	29 (59.18%)	27 (57.45%)	
Yes	40 (41.67%)	20 (40.82%)	20 (42.55%)	
**At Cycle 8**	***N*** **= 88**	***N*** **= 44**	***N*** **= 44**	
Febrile Neutropenia (FN)				NA
No	88 (100.00%)	44 (100.00%)	44 (100.00%)	
Yes	0 (0.00%)	0 (0.00%)	0 (0.00%)	
FN-Associated Hospitalization (days)				NA
*N*	0	0	0	
Mean ± SD	-	-	-	
Median (Inter-quartiles)	-	-	-	
Min, Max	-	-	-	
Dose delays				0.5569
No	85 (96.59%)	42 (95.45%)	43 (97.73%)	
Yes	3 (3.41%)	2 (4.55%)	1 (2.27%)	
No. of days delayed				NA
*N*	3	2	1	
Mean ± SD	7.3 ± 0.6	7.5 ± 0.7	7.0 ± NA	
Median (Inter-quartiles)	7 (7, 8)	8 (7, 8)	7 (7, 7)	
Min, Max	7, 8	7, 8	7, 7	
Dose reduction				0.6700
No	45 (51.14%)	24 (54.55%)	21 (47.73%)	
Yes	43 (48.86%)	20 (45.45%)	23 (52.27%)	

NA = not available; * *p*-value was obtained by Wilcoxon rank-sum nonparametric test or Fisher exact for continuous or categorical variables as appropriate. *p* < 0.05 was considered statistically significant (Bolded).

**Table A2:** Cycle-per-Cycle Outcomes in Lymphoma Cancer Patients.

Per Cycle Analysis	Total	Neupogen^®^	Grastofil^®^	*p*-Value *
**At Cycle 1**	***N*** **= 31**	***N*** **= 17**	***N*** **= 14**	
Febrile Neutropenia (FN)				0.8869
No	29 (93.55%)	16 (94.12%)	13 (92.86%)	
Yes	2 (6.45%)	1 (5.88%)	1 (7.14%)	
FN-Associated Hospitalization (days)				NA
*N*	2	1	1	
Mean ± SD	7.5 ± 0.7	8.0 ± NA	7.0 ± NA	
**At Cycle 2**	***N*** **= 30**	***N*** **= 17**	***N*** **= 13**	
Febrile Neutropenia (FN)				NA
No	30 (100.00%)	17 (100.00%)	13 (100.00%)	
Yes	0 (0.00%)	0 (0.00%)	0 (0.00%)	
FN-Associated Hospitalization (days)				NA
*N*	0	0	0	
Mean ± SD	-	-	-	
Dose delayed				NA
No	30 (100.00%)	17 (100.00%)	13 (100.00%)	
Yes	0 (0.00%)	0 (0.00%)	0 (0.00%)	
No. of days delayed				0.5209
*N*	0	0	0	
Mean ± SD	-	-	-	
Dose reduction				0.8439
No	28 (93.33%)	16 (94.12%)	12 (92.31%)	
Yes	2 (6.67%)	1 (5.88%)	1 (7.69%)	
**At Cycle 3**	***N*** **= 30**	***N*** **= 17**	***N*** **= 13**	
Febrile Neutropenia (FN)				NA
No	30 (100.00%)	17 (100.00%)	13 (100.00%)	
Yes	0 (0.00%)	0 (0.00%)	0 (0.00%)	
FN-Associated Hospitalization (days)				NA
*N*	0	0	0	
Mean ± SD	-	-	-	
Dose delayed				0.3738
No	29 (96.67%)	16 (94.12%)	13 (100.00%)	
Yes	1 (3.33%)	1 (5.88%)	0 (0.00%)	
No. of days delayed				NA
*N*	1	1	0	
Mean ± SD	7.0 ± NA	7.0 ± NA.	-	
Dose reduction				0.4920
No	28 (93.33%)	15 (88.24%)	13 (100.00%)	
Yes	2 (6.67%)	2 (11.76%)	0 (0.00%)	
**At Cycle 4**	***N*** **= 25**	***N*** **= 14**	***N*** **= 11**	
Febrile Neutropenia (FN)				0.3656
No	24 (96.00%)	13 (92.86%)	11 (100.00%)	
Yes	1 (4.00%)	1 (7.14%)	0 (0.00%)	
FN-Associated Hospitalization (days)				NA
*N*	1	1	0	
Mean ± SD	12.0 ± NA	12.0 ± NA	-	
Dose delayed				0.4400
No	24 (96.00%)	14 (100.00%)	10 (90.91%)	
Yes	1 (4.00%)	0 (0.00%)	1 (9.09%)	
No. of days delayed				NA
*N*	1	0	1	
Mean ± SD	21.0 ± NA	-	21.0 ± NA.	
Dose reduction				0.8403
No	20 (80.00%)	11 (78.57%)	9 (81.82%)	
Yes	5 (20.00%)	3 (21.43%)	2 (18.18%)	
**At Cycle 5**	***N*** **= 24**	***N*** **= 13**	***N*** **= 11**	
Febrile Neutropenia (FN)				0.4819
No	22 (91.67%)	11 (84.62%)	11 (100.00%)	
Yes	2 (8.33%)	2 (15.38%)	0 (0.00%)	
FN-Associated Hospitalization (days)				NA
*N*	2	2	0	
Mean ± SD	15.5 ± 2.1	15.5 ± 2.1	-	
Dose delayed				0.3474
No	23 (95.83%)	12 (92.31%)	11 (100.00%)	
Yes	1 (4.17%)	1 (7.69%)	0 (0.00%)	
No. of days delayed				NA
*N*	1	1	0	
Mean ± SD	17.0 ± NA	17.0 ± NA	-	
Dose reduction				0.7721
No	16 (66.67%)	9 (69.23%)	7 (63.64%)	
Yes	8 (33.33%)	4 (30.77%)	4 (36.36%)	
**At Cycle 6**	***N*** **= 22**	***N*** **= 11**	***N*** **= 11**	
Febrile Neutropenia (FN)				NA
No	22 (100.00%)	11 (100.00%)	11 (100.00%)	
Yes	0 (0.00%)	0 (0.00%)	0 (0.00%)	
FN-Associated Hospitalization (days)				NA
*N*	0	0	0	
Mean ± SD	-	-	-	
Dose delayed				NA
No	20 (90.91%)	9 (81.82%)	11 (100.00%)	
Yes	2 (9.09%)	2 (18.18%)	0 (0.00%)	
No. of days delayed				NA
*N*	2	2	0	
Mean ± SD	7.5 ± 0.7	7.5 ± 0.7	-	
Dose reduction				0.6471
No	15 (68.18%)	7 (63.64%)	8 (72.73%)	
Yes	7 (31.82%)	4 (36.36%)	3 (27.27%)	

NA = not available; * *p*-value was obtained by Wilcoxon rank-sum nonparametric test or Fisher exact for continuous or categorical variables as appropriate. *p* < 0.05 was considered statistically significant (Bolded).
